# Retraction Note: Impact of NF-κB pathway on the intervertebral disc inflammation and degeneration induced by over-mechanical stretching stress

**DOI:** 10.1186/s12950-022-00312-z

**Published:** 2022-10-10

**Authors:** Hui Xu, Guobao Qi, Kunpeng Li, Keshi Yang, Dawei Luo, Zhongxu Cai

**Affiliations:** 1 Department of Spinal Surgery, Liaocheng People’s Hospital, No. 67, Dongchang Xilu Road, 252000 Liaocheng, Shandong China; 2 Department of Spinal Surgery, Dongying People’s Hospital, No. 317, Nanyi Road, 257091 Dongying, Shandong China

**Retraction Note**: *J Inflamm***18**, 6 (2021). 10.1186/s12950-021-00273-9

The Editors in Chief have retracted this article. After publication it was noted that there was overlap between Fig. 2 C and Fig. 3 from another previously published article [1]. The Editors in chief have therefore lost confidence in the integrity of the data in this article.

Zhongxu Cai has stated on behalf of all co-authors that they agree to this retraction.
